# Hypertension and Cerebral Microangiopathy (Cerebral Small Vessel Disease): Genetic and Epigenetic Aspects of Their Relationship

**Published:** 2018

**Authors:** L. A. Dobrynina, M. R. Zabitova, L. A. Kalashnikova, E. V. Gnedovskaya, M. A. Piradov

**Affiliations:** Research center of neurology, Volokolamskoe Shosse 80, Moscow, 125367, Russia

**Keywords:** genetics, epigenetics, arterial hypertension, cerebral microangiopathy, small vessel disease, neuroimaging

## Abstract

Hypertension (HT) and its cerebral complications are extremely vexing medical and social problems. Despite the obvious association between hypertension and the clinical and neuroimaging features of cerebral microangiopathy (CMA) (also known as cerebral small vessel disease), the causal links between them remain ambiguous. Besides, antihypertensive therapy as the only way to manage these patients does not always prevent brain damage. Knowledge about the key factors and mechanisms involved in HT and CMA development is important for predicting the risk of cerebral complications and developing new approaches to their prevention and treatment. At present, genome-wide association studies and other approaches are used to investigate the common hereditary mechanisms of HT and CMA development, which will explain a large number of CMA cases not associated with hypertension, lack of a correlation between HT severity and the degree of cerebral injury, and failure of antihypertensive therapy to prevent CMA progression. Epigenetic markers likely play a modulating role in the development of these diseases.

## INTRODUCTION

For many decades, hypertension (HT) has been one of the leading pathologies in the structure of morbidity and mortality in populations across the world. On average, HT is diagnosed in every third adult and has an age-dependent pattern and a tendency to increase in its incidence rate with the next decades [1-4]. The cerebral complications of HT develop very early, dominate, and contribute the most to the structure of HT-associated mortality [5-7]. HT-associated lesions in small cerebral arteries, arterioles, capillaries, and venules lead to the development of the progressive cerebral microangiopathy (CMA) (in Russia, it is included in a broader concept termed dyscirculatory encephalopathy; in the foreign literature, it is referred to as small vessel disease) that is one of the main causes of strokes and dementia [5, 8-14]. At present, there is an understanding of the relationship between white matter hyperintensities (WMH, previously known as leukoaraiosis), which is a recognized neuroimaging marker of CMA, and the duration and profile of HT, cognitive impairments/ dementia, disability, and risk of stroke and death [10, 15-24], as well as the possibility of delaying the progression of WMH and cognitive impairments by using adequate antihypertensive therapy [11, 25, 26]. The latter is implicitly confirmed by the recent Framingham study that demonstrated a reduction in the rate of vascular dementia in subjects with secondary and higher education, which was presumably related to their greater awareness, access to medical care, and adherence to therapy. However, the reduced prevalence of most vascular risk factors in these people, including HT control, could not adequately explain the reduction in the rate of dementia [27]. In addition, the absence of a direct correlation between the severity of HT and the degree of clinical and neuroimaging manifestations of CMA, which is well-known in clinical practice, as well as the risk of CMA in middle-aged and elderly individuals without HT is indicative of the ambiguity of the relationships between CMA and HT and inapplicability of antihypertensive therapy as the only management option for CMA patients to arrest the progression of CMA.

Epidemiological analysis has revealed a high coefficient of heritability of leukoaraiosis in family and twin studies (55–71%) [28], as well as a more than 1/3 (36%) overlap between the hereditary factors controlling pulse pressure and indicators of fractional anisotropy of the white matter [29]. In this regard, the close relationship between HT and CMA may be explained by common genetic disorders.

The influence of common environmental factors, including diet and lifestyle, on the development of both diseases may indicate an involvement of common epigenetic mechanisms of gene expression regulation. This is in line with the Framingham study results [27]. Adherence to a healthy lifestyle and diet, more anticipated among individuals with high education, may be one of the explanations for the reduced dementia in such populations.

## GENETICS OF HT AND CMA

The main aspects of genetic studies of HT and CMA are as follows:

• investigation of monogenic (Mendelian) forms;

• analysis of the candidate genes associated with known signs/mechanisms of disease development; and

• genome-wide association studies (GWAS) – clarification of the nucleotide variants associated with HT and CMA.

**Monogenic forms of HT and CMA**

The number of monogenic (Mendelian) forms of HT and CMA known to date is small. Monogenic forms of HT are characterized by a very low prevalence in the population [30]. Despite pathogenetic heterogeneity, they are all associated with mutations in the components of the renin-angiotensin-aldosterone system (RAAS), which ultimately lead to impairment of urinary sodium excretion. Below, we provide a list of the main genes whose mutations are associated with particular forms of CMA (*Table 1*). The best known monogenic forms of CMA include CADASIL, caused by a mutation in the NOTCH_3_ receptor gene located on chromosome 19q12; CARASIL (mutations in the *HTRA1 *gene encoding serine peptidase 1, which is located on chromosome 10q25); an autosomal dominant form of COL4A1 (the *COL4A1 *gene encoding collagen type IV α1, which is mapped on chromosome 13q34); RVCL (the *TREX1 *gene encoding DNase III exhibiting 3’–5’ exonuclease activity, which occurs on chromosome 3p21); and the Fabry disease (X-linked disease caused by mutations in the *GLA *gene encoding α-galactosidase A, chromosome Xq22) [31]. In all these forms of CMA, an altered protein product leads to a loss of the structural and functional integrity of small arteries with subsequent secondary injury to the brain substance. CADASIL is the most commonly diagnosed hereditary CMA. Its estimated prevalence is 4.6/100,000 in the adult population, and the mutation rate in the *NOTCH_3_*gene is 10.7/100,000 adults [32]. Most CADASIL patients have no HT, but when it is present in carriers of certain polymorphisms in the *NOTCH_3_*gene, the risk of injury to the white matter increases [33]. Accurate information about the prevalence of other monogenic CMAs is unavailable, and HT occurrence has not been analyzed.

**Candidate genes for HT and CMA**

The main directions in the search for the candidate genes associated with individual risk of the disease include the investigation of the genes that are the key components of RAAS, endothelium, hemostasis, inflammation and immune response, neurotrophic factors, etc. (*Table 2*) (the names of the genes/polymorphisms/proteins are given in accordance with international nomenclature https://www.ncbi.nlm.nih.gov/genome/guide/human/).

Apparently, impaired functioning of the RAAS is central in the development of HT. The imbalance in the functioning of RAAS components is associated with vasoconstriction, electrolyte imbalance with sodium and water retention, and vascular remodeling [30]. The role of angiotensinogen (*AGT*), the angiotensin-converting enzyme (*ACE*), and aldosterone synthase (*CYP11B2*) genes in these processes has been the best studied. Studies of the significance of angiotensin II receptor type 1 and 2 (*AGTR1*, *AGTR2*) and renin genes yielded controversial results [34-36].

**The AGT gene of angiotensinogen (chromosome 1q42).**

The *AGT *gene belongs to the serpin gene superfamily, is expressed in the brain, liver, heart, adipose tissue, kidney, and vascular walls, and encodes a precursor of angiotensin II (AGTII) – a physiological regulator of blood pressure (BP) and water-salt metabolism. Among the large number of molecular variants of the *AGT *gene, only the polymorphisms rs699C > T (codon M235T) and rs4762C > T (codon T174M) have been shown to be associated with HT and plasma angiotensinogen levels in Europeans and white Americans [37, 38]. Attempts to reproduce these studies have led to ambiguous results [39, 40].

According to [41], the homozygous genotype of the codon M235T of the *AGT *gene is associated, significantly and independently of HT, to the progression of brain injury in CMA, but not with carotid atherosclerosis, which suggests that this genotype is a genetic marker of progressive brain injury in CMA. However, the number of lacunae detected in the brain of carriers of this genotype was significantly smaller than that in heterozygous carriers [35]. The latter is explained by a probable involvement of angiotensinogen in clotting processes. This is indirectly confirmed by the relationship, independent of increased BP, between an *AGT *gene polymorphism and the development of lacunar infarct [35]. An investigation of polymorphisms C521T (T174M, T207M) and rs699 (T704C) of the *AGT *gene in a Transbaikalian population failed to establish any link with the development of chronic cerebral ischemia (most likely associated with CMA) [42]. An analysis of a group of 410 adults (aged 50–75 years) with CMA-specific changes revealed by MRI demonstrated that the four most common mutations in the gene promoter were combined into haplotypes. In this case, the B-haplotype (*–6:A*, *–20:C*, –153:G, –218:G) was an HT-independent risk factor of changes in the brain. The (B/B) homozygosity of this haplotype was found to increase eight-fold or more the risk of brain injury in CMA [43]. As was shown later, the B haplotype enhances the main transcriptional activity of the *AGT *promoter in astrocytes (the main site of AGT synthesis in the brain), which suggests a potential association between white matter injury and impaired RAAS activity [44]. A later study found no relationship between single polymorphisms in the *ACE *and *AGT *genes and white matter injury in CMA. The exception was the polymorphism –20A > C in the *AGT* promoter region, which was associated with leukoaraiosis in HT patients [45]. Recently, in healthy elderly subjects, a relationship was established between the M268T polymorphism (previously M235T) of the *AGT *gene and a loss of the microstructure of some white matter regions. This was assessed by MRI fractional anisotropy [46].

**Table 1:** Monogenic forms of CMA

Disease	Locus	Gene	Protein	Mode of inheritance	Clinical manifestations	MRI findings	Morphological changes in the vascular wall
CADASIL	19q12	NOTCH3	NOTCH3 receptor	Autosomal dominant	Migraine with aura, lacunar strokes, cognitive impairments/ dementia, mental disorders	WMH in the temporal lobe poles and external capsules, subcortical LIs	Accumulation of osmiophilic deposits
CARASIL	10q25	HTRA1	HtrA, serine peptidase 1	Autosomal recessive	Cognitive disorders, lacunar strokes, alopecia, low back pain	Subcortical LIs	Extensive smooth muscle cell degeneration
COL4A1	13q34	COL4A1	α1-collagen type IV	Autosomal dominant	Porencephaly, infantile cerebral palsies, Axenfeld-Rieger anomaly, nephropathy, cramps, cataract, retinal hemorrhages	WMH, LI, subcortical microhemorrhages	Basement membrane damage
RVCL	3p21	TREX1	DNase III with 3’-5’-exo- nuclease activity	Autosomal dominant	Retinal vasculopathy, migraine, cognitive impairments, mental disorders, Raynaud’s phenomenon, hepatic cirrhosis, nephropathy, osteonecrosis	Subcortical LIs, WMH	Basement membrane damage
Fabry disease	Xq22	GLA	α-galactosi- dase A	X-linked	Angiokeratomas, acroparesthesias, damage to the kidneys and heart, changes in the facial skull	WMH and LI in the vertebral-basilar system, dolichoectasia of the main artery	Accumulation of lysosomal deposits in endothelial and smooth muscle cells

*The ACE gene of the angiotensin-converting enzyme (chromosome 17q23).* The angiotensin-converting enzyme converts angiotensin 1 into a potent angiotensin-2 vasopressor, cleaves bradykinin (stimulant of endothelial NO formation) to inactive metabolites, and regulates the release of aldosterone. HT is better associated with the insertion-deletion (I/D) polymorphism in intron 16 of the *ACE *gene, which is identified by the presence/absence of Alu repeats. A combination of II+ID alleles of the I/D polymorphism with a daily intake of more than 2,300 mg of salt was found to be associated with HT and subsequent obesity [47]. The D allele of I/D polymorphism was found to be associated with diastolic and systolic HT, daily variability of HT, and injury to target organs [48-52]. The D/D genotype was shown to play a significant role in the development of CMA-associated cerebral lesions [53, 54] and in the prediction of the risk of this lesion [55].

**Table 2:** Association of candidate genes with MRI markers of CMA and/or HT

Gene	Polymorphism	Association	
		MRI markers of CMA	HT
AGT	rs699	Lacunes [35, 41], WMH [41] Loss of white matter tract microstructure [46] Not verified [42, 45]	Found [37, 8] Not verified [39, 40]
AGT	rs4762	Not verified [42]	Found [37, 38] Not verified [39, 40]
AGT	–20A > C	WMH [45]	Found [45]
ACE	I/D Alu-sequences	Alu-sequences WMH [52–55] Not verified [46]	Found [47-52]
CYP11B2	rs1799998	WMH, expansion of perivascular spaces [36, 56]	No data
NOS1	rs3782218	Contradictory data	Found [59]
NOS3	rs3918226	Contradictory data	Found [59]
NOS3	rs3918227	Contradictory data	Found [59]
EDN1	rs5370	Not verified [45]	Found [63, 64]
MTHFR	rs1801133	WMH, LI [65, 66] Not verified [55, 67, 68]	Found [65, 66]
PLAT	rs2020918	LI [71, 72]	No data
FGB	rs1800790	LI [73]	No data
IL1B	rs16944	LI [78], WMH [81]	No data
IL6	rs1800795	LI [79], WMH [80]	Not verified [74]
IL6	rs1800796	LI [80]	Found [75]
TNFA	rs1800629	No data	Found [75]
MMP2	rs243865	WMH [85]	No data
MMP2	rs1030868 rs2241145 rs2287074 rs2287076 rs7201	WMH [84]	No data
MMP9	rs3918242	No data	Found [78]
CRP	rs3091244	WMH [81]	No data
VEGF	rs2010963	LI [86]	Found [86]
BDNF	rs6265	WMH [87]	No data

*The aldosterone synthase gene (CYP11B2, chromosome 8q24.3).* Aldosterone synthase catalyzes the synthesis of aldosterone from deoxycorticosterone. Aldosterone increases tubular reabsorption of Na^+^ and elimination of K+, which enhances the ability of tissues to retain water. The protective effect of the C allele of polymorphism rs1799998 (–344T > C) of *CYP11B2 *is manifested in leukoaraiosis and the expansion of perivascular spaces [36, 56].

**Genes affecting endothelial function**

These genes include the *NOS1 *gene encoding neuronal NO synthase (nNOS) (chromosome 12q24.2-q24.3) and the *NOS3 *gene of endothelial NO synthase (eNOS) (chromosome 7q35-q36). NO plays an important role in maintaining the homeostasis of the endothelium. Impairment of NO production leads to failure of physiological vasodilation, increased aggregation and adhesion of platelets, proliferation and migration of smooth muscle cells, and inflammation – the main pathophysiological mechanisms of HT and CMA. Inhibition of the *NOS1 *gene in the medulla oblongata and hypothalamus is associated with the pathogenesis of systemic hypertension [57]. A genome-wide association study for schemic stroke risk factors iditified *NOS1 *as a potential candidate gene [58]. Among the 58 single nucleotide substitutions in the *NOS *genes associated with HT, there were the polymorphic sites rs3782218 in *NOS1 *and rs3918226 and rs3918227 in *NOS3 *[59]. The data published by Russian researchers who have studied the association between the polymorphisms rs1799983 (G298A, G894T) and 4a/4b of the *NOS3 *gene and HT and the remodeling of the wall of large cerebral arteries are contradictory [60-62]. There are no stably reproducible results concerning the significance of *NOS *polymorphisms in the development of CMA.

*The endothelin-1 gene (EDN1, chromosome 6p24.1). *The polymorphism rs5370G> T (codon K198N) of the *EDN1 *gene encoding a potent vasoconstrictor, endothelin has been shown to be associated with the development of HT [63, 64]. The role of the polymorphisms in endothelin and endothelin receptor genes in CMA has not been verified [45].

*The methylenetetrahydrofolate reductase gene (MTHFR, chromosome 1p36.22).* Methylenetetrahydrofolate reductase is involved in the conversion of homocysteine to methionine in the presence of cofactors (vitamins B6 and B12) and a substrate (folic acid). Most population-based studies have confirmed the association between the polymorphism rs1801133C > T (C677T) in the *MTHFR *gene and hyperhomocysteinemia and CMA (WMH volume or lacunar infarcts) in patients with and without HT [65, 66]. However, a significant number of studies have found no evidence of association between the polymorphism C677T and WMH [55, 67, 68]. Studies that have evaluated the total effect of carriage of several polymorphisms have revealed significant worsening of CMA (WMH, lacunar infarcts) when a C677T variant of the *MTHFR *gene was combined with the D/D genotype of the I/D polymorphism of the *ACE *gene. At the same time, combination of one of the genotypes 2/2, 2/3, 4/4, or 4/3 of the *APOE *gene (chromosome 19q13, apolipoprotein E) with the *MTHFR *C677T or *ACE *D/D genotype can act as an independent genetic risk factor for leukoaraiosis [69, 70].

**Genes of the hemostasis system**

There is contradictory data on the association between the polymorphism rs2020918 (–7351C/T) in the tissue plasminogen activator gene (*PLAT*, or *TPA*) and the development of lacunar infarcts [71, 72]. The AA genotype of polymorphism rs1800790 (455G/A) of the fibrinogen gene (*FGB*) has been demonstrated to be associated with an increased risk of multiple lacunar infarcts [73].

**Genes of immune response and inflammation**

The largest number of studies that have explored the pathogenetic significance of mutations in the genes of inflammation are devoted to genetic variants of cytokines. The polymorphisms rs1800796 (572G > C) of the interleukin-6 gene (*IL6*) [74] and rs1800629 (308G > A) of the *TNFα *gene encoding tumor necrosis factor alpha have been found to be associated with the development of HT in the Asian population [75]. *TNFα *gene polymorphisms also affect the course of HT in the population of the Russian Central Chernozem Region [76]. The polymorphism rs3918242 (–1562C > T) in the matrix metalloproteinase-9 gene (*MMP9*) is associated with a risk of HT [77].

A study of the polymorphism rs16944 (–511C > T) in the interleukin-1β gene (*IL1B*) revealed a prevalence of the T/T genotype in patients with lacunar strokes compared to other stroke subtypes. An analysis with allowance for concomitant factors demonstrated that the T/T genotype of the *IL1B *gene (polymorphism 511C > T) was an independent risk factor of strokes in CMA [78]. The C/C genotype of the polymorphism rs1800795 (–174G > C) of the *IL6 *gene has been shown to be associated with lacunar stroke and an increased risk of WMH [79, 80], and the polymorphism rs1800796 (–572G > C) has been associated with the development of asymptomatic infarcts [80]. Worsening of WMH was detected in old and elderly subjects without neurological deficiency, homozygous carriers of the *IL1B *rs16944 (–511C > T) T allele and T allele at position –286 (rs3091244) of the C-reactive protein gene (*CRP*) [81]. There is an increase in the carriage rate of homozygous variants of polymorphisms –31CC of the *IL1B *gene, –174GG of the *IL6 *gene, –197AA of the *IL17A *gene, and –166ArgArg of the *IL17F *gene, as well as the alleles IL-1β-31C of the *IL1B *gene and IL17F- 166Arg of the *IL17F *gene in elderly patients (Transbaikalian population of Russia) with chronic ischemia most likely associated with CMA [82]. No association of the haplotypes/polymorphisms of the *CRP *gene with CMA has been detected [83]. It has been demonstrated that carriage of the allelic variants rs1030868:g.T, rs2241145:g.C, rs2287074:g.A, rs2287076:g.C, and rs7201:g.C of the *MMP2 *gene is associated with a risk of lacunar infarcts; the rs7201:gC allele is an independent risk factor for their development [84]; and the C/C genotype of the *MMP2 *gene (rs243865, 1306T > C) plays an independent predictive role in the development of leukoaraiosis [85].

**Genes of trophic factors**

The polymorphism rs2010963 (–634G > C) of the vascular endothelial growth factor gene (*VEGF*) was found to be associated with the development of lacunar stroke in [86]. The multidirectional effect of homozygous carriage of various Val66Met (rs6265) codon alleles of the brain-derived neurotrophic factor gene (*BDNF*) in WMH and cognitive disorders has been revealed: the Met allele is protective, and the Val allele is damaging [87].

Therefore, a significant number of genes selected based on data on the causes and mechanisms of disease development have been studied to date. The identified and reproducible associations of polymorphisms of these genes with HT and CMA suggest that these polymorphisms are risk factors for the diseases. However, most researchers have indicated that the cumulative data explain only a small part of HT and CMA cases and their course manifestations, and that the rather frequent contradictions in the results are due to the impossibility of reproducing them in other samples [88]. The data on an HT-independent association of mutations in the key pathogenetic factors, e.g., the TT genotype (M235T polymorphism) and B haplotype of the *AGT *gene, with the development and progression of CMA confirm the ambiguity of the relationships between CMA and HT. In addition, the dependence of the clinical significance of mutations on environmental factors, in particular the development of HT and obesity in carriers of the *ACE *gene I/D polymorphism and the effect of a high daily salt uptake [47], confirm the need for a clarification of the effect of environmental factors on gene expression when studying the mechanisms of HT and CMA. An approach based on an investigation of candidate genes has some limitations in assessing the various possible variants of interaction and overlap of inherited features.

## GENOME-WIDE ASSOCIATION STUDIES (GWAS)

To date, several genome-wide studies aimed at clarifying the loci associated with HT and CMA have been performed (*Table 3*). However, only a few of these studies have identified loci with genome-wide significance (*p * < 5 × 10^-8^).

The Global Blood Pressure Genetics (BPGen) consortium and the Cohorts for Heart and Ageing Research in Genome Epidemiology (CHARGE) consortium analyzed 34,433 and 29,136 individuals, respectively, and identified eight loci associated with HT, three of which were common to both studies [89, 90]. Later, the International Consortium for Blood Pressure Genome-Wide Association Studies (ICBP GWAS) analyzed the data of 200,000 individuals and identified 29 loci, six of which had been previously identified as significant for HT [91]. Many of these loci are considered as potential candidates, including the *NPPA *and *NPPB *genes encoding natriuretic peptides.

In 2011, the CHARGE consortium performed the first genome-wide association study for WMH in 9,361 stroke-free individuals of European descent (mean age 69.5 years; 42.6% males) [92]. Six single-nucleotide polymorphisms (with a genome-wide level of significance) associated with a high risk of WMH were identified. Of these, rs3744028 in the *TRIM65 *gene had the most significant association with WMH severity. The polymorphisms were mapped to a single genetic locus – a 17q25 chromosome region containing seven major genes: *WBP2*, *TRIM65*, *TRIM47*, *MRPL38*, *FBF1*, *ACOX1*, and *UNC13D*. All these genes are involved in the processes of neuroinflammation and immune system functioning. The results of this meta-analysis could be reproduced in subsequent studies [93-95].

In 2015, the results of a multi-ethnic genome-wide association study for WMH were published [96]. The study involved 21,079 middle-aged dementia- and stroke-free individuals selected from 29 population-based cohorts: European (n=17,936), African (n=1,943), Hispanic (n=795), and Asian (n=405). The obtained data confirmed the association of a 17q25 chromosome region with a risk of WMH; also, three loci (chromosomes 10q24, 1q22, and 2p16.1) associated with WMH in more than one population were identified. The genetic loci regulating systolic and diastolic HT were shown to be associated with the development of WMH. A new chromosome 10q24 locus containing genome-wide significant polymorphisms in the introns of the *PDCD11*, *NEURL*, *SH3PXD2A*, *TAF5*, and *CALHM1 *genes is associated with the development of brain tumors (medulloblastomas, astrocytomas, gliomas), with *CALHM1 *being associated with both the regulation of calcium homeostasis and formation of amyloid beta. Earlier, the polymorphism rs2984613 (chromosome 1q22; *PMF1 *and *SLC25A44 *genes) had been found to be associated with non-lobar intracerebral hemorrhage [97].

In 2015, the results of a genome-wide association study for white matter lesion progression in elderly individuals of European descent were also published [98]. Progression of white matter lesions was observed in 1,085 (14%) participants in the study, which was indicative of the low significance of genetic factors in the progression of this lesion in the elderly. The findings were explained by a possible omission of significant polymorphisms, a relatively short follow-up period to assess the contribution of genetic factors to progression, and the age of the study participants. A potential role of genetic factors in white matter lesion progression in young people was suggested.

**Table 3:** Results of genome-wide association studies (GWAS) for HT and CMA

Study	Single nucleotide polymorphism	Locus	P-value	
			Systolic BP	Diastolic BP
HT				
Global BPGen (Global Blood Pressure Genetics) [89] Sample size – 34,433 individuals	rs17367504 rs11191548 rs12946454 rs16998073 rs1530440 rs653178 rs1378942 rs16948048	1p36^*^ 10q24^*^ 17q21 4q21 10q21 12q24^*^ 15q24^*^ 17q21	1 × 10^-5^ 3 × 10^-17^ 4 × 10^-6^	7 × 10^-9^ 3 × 10^-6^ 1 × 10^-7^ 6 × 10^-8^ 5 × 10^-6^
CHARGE (The Cohorts for Heart and Ageing Research in Genome Epidemiology) [90] Sample size – 29,136 individuals	rs1004467 rs381815 rs2681492 rs2681472 rs3184504 rs9815354 rs11014166 rs2384550 rs6495122	10q24^*^ 11p15 12q21 12q21 12q24 3p22 10p12 12q24^*^ 15q24^*^	1.99 × 10^-6^ 5.76 × 10^-7^ 3.01 × 10^-11^ 5.73 × 10^-7^	3.74 × 10^-8^ 1.68 × 10^-8^ 7.88 × 10^-7^ 8.82 × 10^-7^ 1.32× 10^-7^ 8.10 × 10^-7^
ICBP GWAS (International Consortium for Blood Pressure Genome-Wide Association Studies) [91] Sample size – 200,000 individuals	rs2932538 rs13082711 rs419076 rs13107325 rs13139571 rs1173771 rs11953630 rs1799945 rs805303 rs4373814 rs932764 rs7129220 rs633185 rs2521501 rs17608766 rs1327235 rs6015450 rs17367504 rs3774372 rs1458038 rs1813353 rs4590817 rs11191548 rs381815 rs17249754 rs3184504 rs10850411 rs1378942 rs12940887	1p13 3p24 3q26 4q24 4q32 5p13 5q33 6p22 6p21 10p12 10q23 11p15^*^ 11q22 15q26 17q21 20p12 20q13 1p36^*^ 3p22^*^ 4q21 10p12 10q21^*^ 10q24 11p15^*^ 12q21 12q24^*^ 12q24^*^ 12q24^*^ 17q21^*^	1.2 × 10^-9^ 1.5 ×10^-6^ 1.8 × 10^-13^ 3.3 × 10^-14^ 1.2 × 10^-6^ 1.8 ×10×^-16^ 3.0 × 10^-11^ 7.7 × 10^-12^ 1.5 × 10^-11^ 4.8 × 10^-11^ 7.1 × 10^-16^ 3.0 × 10^-12^ 1.2 × 10^-17^ 5.2 × 10^-19^ 1.1 × 10^-10^ 1.9 × 10^-8^ 3.9 × 10^-23^ 8.7 × 10^-22^ 0.39 1.5 × 10^-23^ 2.6 × 10^-12^ 4.0 × 10^-12^ 6.9 × 10^-26^ 5.3 × 10^-11^ 1.8 × 10^-18^ 3.8 × 10^-18^ 5.4 × 10^-8^ 5.7 ×10^-23^ 1.8 × 10^-10^	9.9 × 10^-10^ 3.8 × 10^-9^ 2.1 × 10^-12^ 2.3 × 10^-17^ 2.2 × 10^-10^ 9.1 × 10^-12^ 3.8 × 10^-11^ 1.5 × 10^-15^ 3.0 × 10^-11^ 4.4 × 10^-10^ 8.1 × 10^-7^ 6.4 × 10^-8^ 2.0 × 10^-15^ 1.9 × 10^-15^ 0.017 1.4 × 10^-15^ 5.6 × 10^-23^ 3.5 × 10^-19^ 9.0 × 10^-14^ 8.5 × 10^-25^ 2.3 × 10^-15^ 1.3 × 10^-12^ 9.4 × 10^-13^ 5.3 × 10^-10^ 1.2 × 10^^-14^^ 3.6 × 10^-25^ 5.4 × 10^-10^ 2.7 × 10^-26^ 2.3 × 10^-14^
WMH				
CHARGE (The Cohorts for Heart and Ageing Research in Genome Epidemiology) [92] Sample size – 9,361 individuals	rs3744028 rs1055129 rs3744017 rs936393 rs9894383 rs11869977	17q25	4.0 × 10^-9^ 4.1 × 10^-8^ 7.3 × 10^-9^ 6.8 × 10^-9^ 5.3 × 10^-9^ 5.7 × 10^-9^	
Multi-ethnic GWAS [96] Sample size – 21,079 individuals	rs72848980 rs7894407 rs7214628 rs12357919 rs7909791 rs78857879 rs2984613 rs11679640	17q25^*^ 17q25^*^ 17q25 10q24.33 10q24.33 2p16.1 1q22 2p21	2.6 × 10^-9^ 2.6 × 10^-8^ 5.1 × 10^-9^ 1.5 × 10^-8^ 2.9 × 10^-9^ 1.5 × 10^-8^ 2.0 × 10^-8^ 2.1 × 10^-6^	

^*^Loci identified in different studies.

Thus, the performed genome-wide association studies for WMH and HT have revealed the loci whose genes are associated with both WMH and HT variability. Understanding the biological functions of these genes and evaluating their overlap and interaction will shed light on the molecular mechanisms of WMH, their relationships with HT, and their involvement in aging and the development of degenerative lesions. The results thus obtained confirm the multifactorial character of diseases and the dependence of their course on combinations of and interaction between the factors. However, at present, the cumulative effect of the GWAS-identified HT loci explains less than 3% of clinically significant HT variability [99].

## EPIGENETICS OF HT AND CMA

Epigenetic studies of HT and CMA are not numerous and are mainly devoted to the influence of potentially modifiable environmental factors, lifestyle, and diet on gene expression [100].

The main mechanisms of epigenetic modulation of gene expression include DNA methylation, chromatin modifications (including histones), and microRNA regulation [101, 102, 103].

**DNA methylation**

Methylation regulates gene activity through attaching a methyl group to DNA cytosine bases, which disrupts RNA synthesis and, accordingly, translation. Functionally, hypermethylation should lead to the silencing of the gene, and hypomethylation should lead to gene activation [100].

Protein deficiency in the diet of pregnant rats was found to lead to hypomethylation of the *ACE *gene promoter, causing a predisposition to both HT and cognitive impairment in rat offspring [104]. An inverse correlation between *ACE *gene methylation, angiotensin converting enzyme activity, and systolic BP is observed in children. In this case, the methylation level in children with the DD genotype and low weight is significantly lower than that in children with normal weight [105]. As was demonstrated in a Chinese population, the risk of HT depends on the methylation level of the CpG1 and CpG2-5 sites of the *ADD1 *gene (adducin) in females and males, respectively, as well as those of the *ADD1 *promoter regardless of gender [106]. Hypermethylation of the *HSD11B2 *gene promoter (hydroxysteroid 11-beta dehydrogenase 2) leads to impaired conversion of cortisol to cortisone, an increased tetrahydrocortisol/tetrahydrocortisone ratio (active metabolites of cortisol and cortisone), and the development of HT in humans [107, 108]. Hypomethylation of the *NKCC1 *gene (Na-K-2Cl cotransporter-1) in hypertensive rats is associated with increased NKCC1 activity and the development of HT [109]. A relationship between the methylation and expression of the *NET *gene (norepinephrine transporter) in patients with HT and panic attacks was established [110].

The only microarray analysis of DNA methylation in CMA revealed differently methylated genes associated to the onset and progression of leukoaraiosis. For example, the *NDRG1 *gene located on chromosome 8q24 was hypermethylated (the cytoplasmic protein encoded by this gene is involved in the protection of the myelin sheath in the peripheral nervous system, cell differentiation, tumor metastasis and hypoxia, inflammatory response, etc.) and the *BRUNOL4 *gene or the *CELF4 *gene (chromosome 18q12, BRUNO-like 4 protein involved in mRNA stabilization) was hypomethylated compared to the control group with a normal neuroimaging picture [111].

**Histone modification**

Histones (H1/H5, H2A, H2B, H3, and H4) are the main chromatin proteins involved in the packaging of DNA into nucleosomes in the nucleus. The involvement of histones in the mechanisms of epigenetic regulation of nuclear processes is enabled by a 20-amino-acid mobile N-terminal fragment (tail) of the nucleosome. Modification of the N-terminal fragment, which involves various enzymes (arginine methylation, lysine acetylation, serine and threonine phosphorylation, ubiquitination, etc.), affects the interaction between histones and DNA. Dissociation of the histone makes DNA packing less dense and more accessible to regulatory proteins, which leads to an increase in gene activity, while denser packaging reduces gene activity. For example, acetylation of histones enhances transcription, while deacetylation inhibits it; methylation of lysine inhibits transcription, and methylation of arginine activates transcription; hypermethylation or monomethylation of lysine can have the opposite effect – turn off or activate target genes [112].

The enzymatic cascades triggered by aldosterone upon hypomethylation of histone H3 Lys79 are associated with activation of the epithelial Na^+^ channel gene promoter, which leads to an increase in the number of Na channels in the distal parts of the nephron, enhanced cAMP-mediated reabsorption of sodium, and development of HT [113]. Hypermethylation of histone H3 caused by deficiency in lysine-specific demethylase 1 (LSD-1) leads to the development of HT in mice on a high-salt diet [114]. A transgenic mouse model was used to demonstrate the possibility of triggering sympathetic activation upon primary stimulation of B2-adrenergic receptors via the acetylation of histones H3 and H4, followed by turning-off of the *WNK4 *gene promoter (serine-threonine kinase). This led to overexpression of the Na^+^Cl– cotransporter and epithelial Na^+^ channels, sodium reabsorption, and the development of HT [115]. Acetylation of H3 in area postrema neurons was shown to be associated with a change in the sensitivity of the catecholaminergic neurons of the medullary cardiovascular center, followed by sympathetic brainstem activation and HT [116].

**MicroRNA regulation**

MicroRNAs (miR) are endogenous noncoding RNAs ~22 nucleotides in length that regulate gene activity both at the transcriptional level, preventing the transfer of information from DNA to mRNA, and at the translation stage, which leads to a destruction of synthesized mRNA. At least 30% of human genes are believed to be regulated by microRNAs [117]. Activation of miR-21 expression by angiotensin II in human adrenal gland cells was shown to result in increased aldosterone secretion and enhanced cell proliferation [118]. MiR-124 and miR-135a influence the expression of the mineralocorticoid receptor gene *NR3C2 *involved in the mechanisms of familial hypertension and renal salt balance maintenance [119]. An increase in the miR-320 and miR-26b levels and a decrease in the miR-21 level are observed in salt-sensitive Dahl rats. A presumed target of miR-320 is the insulin-like growth factor-1 receptor. The vascular remodeling observed in a high-salt diet is believed to be associated with inactivation of these receptors [120]. There is a relationship between miR-143, miR-145, miR-21, miR-133, and miR-1 and changes in vascular smooth muscle cells and vascular bed remodeling in HT. The peripheral blood mononuclear cells in HT patients are characterized by a low level of miR- 143, miR-145, and miR-133 and a high level of miR- 21 and miR-1 compared to those in a control group. A correlation between the expression of miR-143, miR- 145, and miR-133 and daily diastolic BP in HT [121] has been shown. As demonstrated in a culture of human adrenal cortex cells, miR-24 participates in the epigenetic regulation of aldosterone and cortisol synthesis via action on 11β-hydroxylase (CYP11B), the key enzyme in the synthesis of these hormones [122].

A study of the role of microRNA in the development of leukoaraiosis in CMA [111] revealed eight differentially expressed microRNAs associated with the regulation of pathogenetic gene activity and the molecular mechanisms of leukoaraiosis. It should be noted that the significance of these putative pathogenetic genes was confirmed by the results of GWAS (*TRIM65*, *ACOX1*), searches for candidate genes (*AGTR2*, *MTHFR*, *BDNF*, *MMP3*, *MMP13*), gene expression profiles (*CCR5*, *IL6*, *MAF*, *CALM1*, *COL24A1*, *EPHB2*, *MAP1B*, *CYB5A*, *CDC6*, *CTSC*), and epigenetic studies (*HLA*-*DQA1*, *TGFBR3*, *CD80*, *WDR41*, *RNF39*, *KIAA1199*, *AAK1*). Most of these genes are associated with inflammation in the CNS [111].

The study of the role of the epigenetic dysregulation mechanisms that lead to impaired gene expression in HT and CMA is at its very inception. Given their direct association with cerebrovascular risk factors, epigenetic mechanisms could be thought to play a significant role in the development of age-dependent multifactorial diseases. The probability of such a suggestion is enhanced by the overlap between the results of epigenetic studies and the data of studies of candidate genes, genome-wide studies on the significance of inflammation, the immune response, and RAAS components in the development of CMA. It is crucial to clarify the pathogenetically important epigenetic markers in the blood and cerebrospinal fluid and the possibility of using them to assess injury to the brain and small vessels, given the impossibility of direct intravital visualization and the importance of indirect methods for assessing progressive injury to the brain and small vessels. The topicality of studying epigenetic dysregulation mechanisms has to do not only with the high social impact of the diseases, which only increases as a population tends to live longer, but also with their potential reversibility due to the association with modifiable cerebrovascular risk factors. We suggest that microRNA-regulated processes play an important role in the development of CMA. Our suggestion is based on the leading role played by endothelial disorders in the development of CMA [10] and on the dependence of the expression of major endothelial function regulators on the activity of Dicer ribonuclease and, respectively, microRNA [123].

## CONCLUSION

Investigations of the genetic basis of HT and CMA have identified new molecular targets that could be potentially important in understanding the pathogenetic mechanisms of these diseases and how to correct them therapeutically. However, it is impossible at this point to explain why, given the obvious role of heredity, genetic data do not fully explain the patterns of predisposition to these diseases or do not allow one to predict their development. One of the approaches that could be explored in order to resolve this contradiction may be to investigate the interactions between gene-metabolic and other regulatory networks and the genes associated with the studied diseases. The immediate objective should be the search for epigenetic markers that are associated with various variants of the course of HT and CMA, which would enable to differentiate significant environmental factors at the individual level. This would become the basis for the search for new approaches to the prevention and treatment of these diseases. Reproducibility of results may be achieved through the formation of homogeneous groups of patients and use of uniform standards for assessing brain injury (neuroimaging phenomena and terms) and methods of laboratory diagnostics and post-processing.

## Abbreviations

HT: hypertension
CMA: cerebral microangiopathy
WMH: white matter hyperintensities
LI: lacunar infarct
GWAS: genome-wide association study
RAAS: renin-angiotensin-aldosterone system
CADASIL: cerebral autosomal dominant arteriopathy with subcortical infarcts and leukoencephalopathy
CARASIL: cerebral autosomal recessive arteriopathy with subcortical infarcts and leukoencephalopathy
RVCL: retinal vasculopathy with cerebral leukodystrophy
